# Ancient mitochondrial DNA analysis of human remains in the era of next- generation sequencing

**DOI:** 10.1016/j.jgeb.2026.100828

**Published:** 2026-09-29

**Authors:** Dina W. Ibrahim, Haidan Mostafa El-Shorbagy, Ismail A. Abdelhamid, Yehia Z. Gad

**Affiliations:** a Ancient DNA Lab, National Museum of Egyptian Civilization, Cairo, Egypt; b Zoology Department, Faculty of Science, Cairo University, Giza, Egypt.; c Faculty of Biotechnology, October University for Modern Science and Art, 6 October, Giza, Egypt; d Chemistry Department, Faculty of Science, Cairo University, Giza 12613, Egypt; e Department of Medical Molecular Genetics, Division of Human Genetics and Genome Research, National Research Centre, El-Behooth St., Dokki, Giza 12622, Egypt

**Keywords:** Ancient mitochondrial DNA, mtDNA variations, Miscoding lesions, Paleogenetics, And Bioinformatic tools

## Abstract

The analysis of ancient DNA (aDNA) has become important in recent years, providing new insights on human evolution and population history. Since the emergence of ancient DNA analysis in the mid-eighties, mitochondrial DNA (mtDNA) analysis has been an essential tool in paleogenomic research. Compared to nuclear DNA, its small, circular, multicopy genome is collectively less susceptible to chemical damage and fragmentation and has more potential for retrievability. Despite its longevity, mtDNA's maternal inheritance with no recombination allows researchers to reconstruct maternal lineages and thus partially track human ancestry and migratory patterns. The accuracy and authenticity of mtDNA data retrieved from paleontological and archaeological remains have improved significantly over the past three decades, driven by advances ranging from enhanced contamination control to next-generation genome sequencing and target enrichment. The current review highlights the challenges of recovering and authenticating ancient mtDNA, including DNA fragmentation, postmortem DNA damage, contamination, heteroplasmy, and nuclear mitochondrial sequences (Numts), which can complicate research results. Within this context, the review addresses the rapidly changing sequencing technologies and the use of specialized bioinformatics software that tackle these challenges and enable researchers to reconstruct almost complete mtDNA genomes from human remains. Although mtDNA analysis has limitations and provides only a partial view of the human genomic architecture, it remains an important tool for analyzing maternal ancestry and generating authenticated genomic information of ancient human populations.

## 1 Introduction

The first ancient DNA extract from biological remains was performed by Higuchi & his colleagues in 1984. They examined the dried muscle of the quagga, a zebra-like species (*Equus quagga)* that went extinct in 1883. They were able to obtain many clones from the very low molecular weight DNA of quagga, and its mitochondrial DNA (mtDNA) was also sequenced.^1^ The quagga publication encouraged many researchers to study DNA in other human and non-human remains.

The genetic material recovered from ancient samples revealed a typical pattern of damage at read termini, low copy numbers of endogenous aDNA molecules, high fragmentation, and the presence of exogenous DNA.^2^ The high fragmentation is due to degradation over time, while the typical damage at read termini results from hydrolysis and oxidation that affect the biological sample. These factors influence the accurate reconstruction of the endogenous ancient genome.

The rapid development of automated DNA sequencing technology has revolutionized the field of human archaeogenetics by moving from analyzing parts of the mitochondrial DNA (mtDNA) genome in ancient samples to reconstructing the complete mtDNA genome in the early 2000s.^3^, ^4^, ^5^, ^6^ This was followed by analyzing the whole-genomic data of several ancient individuals.^7^, ^8^, ^9^

Over the past few years, several aDNA studies have provided insights into the historical migration paths and relationships between various human populations, shedding light on the large-scale population dynamics of our ancestors.^10^, ^11^, ^12^, ^13^ Such information is crucial for reconstructing the investigated pedigrees and understanding human evolution, thereby answering many historical questions.^14^

Practically speaking, this kind of data might also be a valuable resource for “storytelling” in outreach, science communication, and museum exhibits. The current review aims to address the use of ancient mitochondrial DNA analysis in the era of next-generation sequencing (NGS) and the generation of whole-genomic data for ancient populations.

## 2 Mitochondrial DNA: structure and characteristics

### 2.1 Overview of the mitochondrial genome

The mitochondrion is an organelle that has its circular genomic DNA (Margulis, 1970) (Fig. 1). A human cell can contain several thousand copies of a small circular mitochondrial DNA (mtDNA).^15^, ^16^ Human mtDNA includes about 16,600 base pairs and encodes 13 proteins that are required for oxidative phosphorylation.

**Fig. 1:**
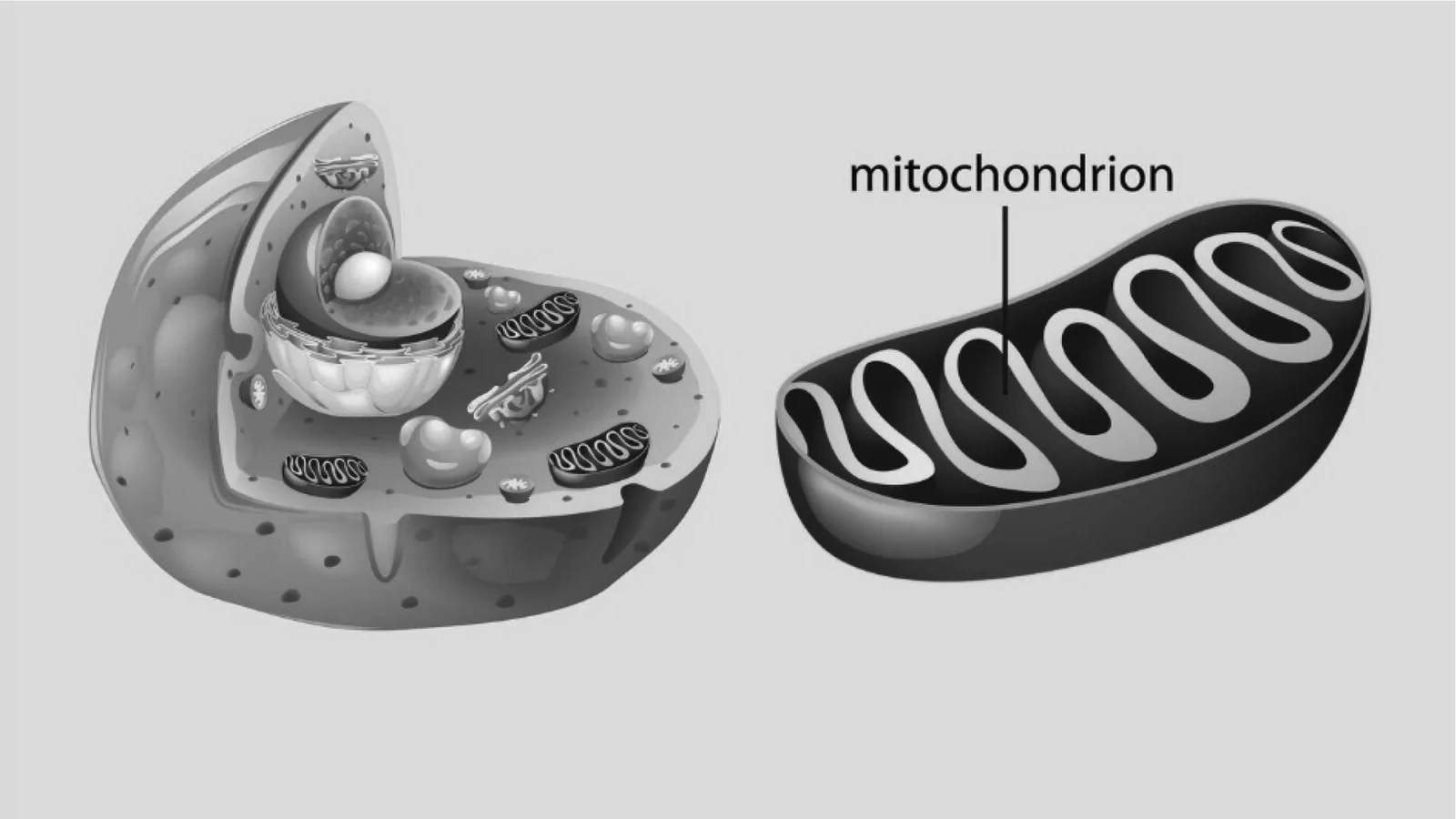
**Structure of the human mitochondrion.** Schematic illustration of the human mitochondrion showing the major structural components of the human mitochondrion.

Most of the mitochondrial genome is coding DNA, unlike nuclear DNA (nDNA), which contains non-coding regions involved in alternative splicing, gene regulation, and other less well-known functions.^17^, ^18^, ^19^ Consequently, mutations in mtDNA often influence function, thus compromising mitochondrial performance. The intron-free structure of mtDNA simplifies mitochondrial gene regulation, which is governed by transcription factors and epigenetic modifications. Understanding these regulatory processes is essential for assessing the impact of mtDNA variants on complex traits or diseases.^19^, ^20^

Being highly abundant per cell and maternally inherited with little or no recombination, mtDNA is believed to be a good representative of the maternal lineages in ancient remains.^21^, ^22^, ^23^ Thus, it can partially be used as a molecular marker to study genetic flow & phylogeny.^24^, ^25^

### 2.2 mtDNA Mutation Rates, Haplogroups, and Variants

#### 2.2.1 Mutation rates

Although mtDNA is present in hundreds to thousands of copies per cell according to cell type, the sequence evolution rate of mtDNA genes is quite rapid, possibly 10–20 times that of comparable nuclear DNA (nDNA) genes.^26^, ^27^, ^28^ This is the result of multiplying a mtDNA mutant fixation rate by a mutation rate, which is possibly 100–1000 times higher than that of nDNA genes: E = μF (where E is the rate of sequence evolution, μ is the mutation rate, and F is the fixation rate).^28^

The mutation rate of mtDNA is higher than that of the nuclear genome due to the lack of proofreading enzymes. So, mtDNA is highly susceptible to mutations, including pathogenic mutations.^19^, ^28^, ^29^ However, not all mtDNA variations are highly deleterious due to their haploid inheritance and their small effective population size.^30^ In addition, highly deleterious variants are removed by negative selection within populations, which is not fully understood, but prevents their existence in the mtDNA of an organism.^31^, ^32^, ^33^, ^34^

#### 2.2.2 Haplogroup nomenclature

Mitochondrial haplogroups consist of sets of related haplotypes defined by distinct combinations of mtDNA single-nucleotide polymorphisms (SNPs) relative to a reference sequence, each originating from a common ancestor.^19^, ^35^ Haplogroup nomenclature consists of a combination of numbers and letters. The macro-haplogroup “L” includes haplogroups L0-L5 and their sub-haplogroups and represents the most ancient African mtDNA sequences, dating back to 130,000–200,000 years before present (YBP). About 65,000 YBP. Haplogroup L3 gave rise to the macrohaplogroups M and N. Those migrated out of Africa, where macrohaplogroup M moved to Asia, generating M1-M80, including C, D, G, and Z. Macrohaplogroup N also moved to Asia, generating A, B, F, and O, but also moved towards Europe, generating I, X, W, and R. The haplogroup R gave rise to the remaining European haplogroups H, V, J, U, and T. Then, haplogroups A, C, and D, which originated in northeastern Siberia, reached America (∼ 20,000 YBP). Also, recent migrations to America brought haplogroups B and X.^28^, ^36^ Nowadays, more than 5400 haplogroups and subgroups are described in the phylogenetic tree, established by van Oven et al. which is updated and available online at http://www.phylotree.org (build 17)^37^ (Fig. 2).

**Fig. 2:**
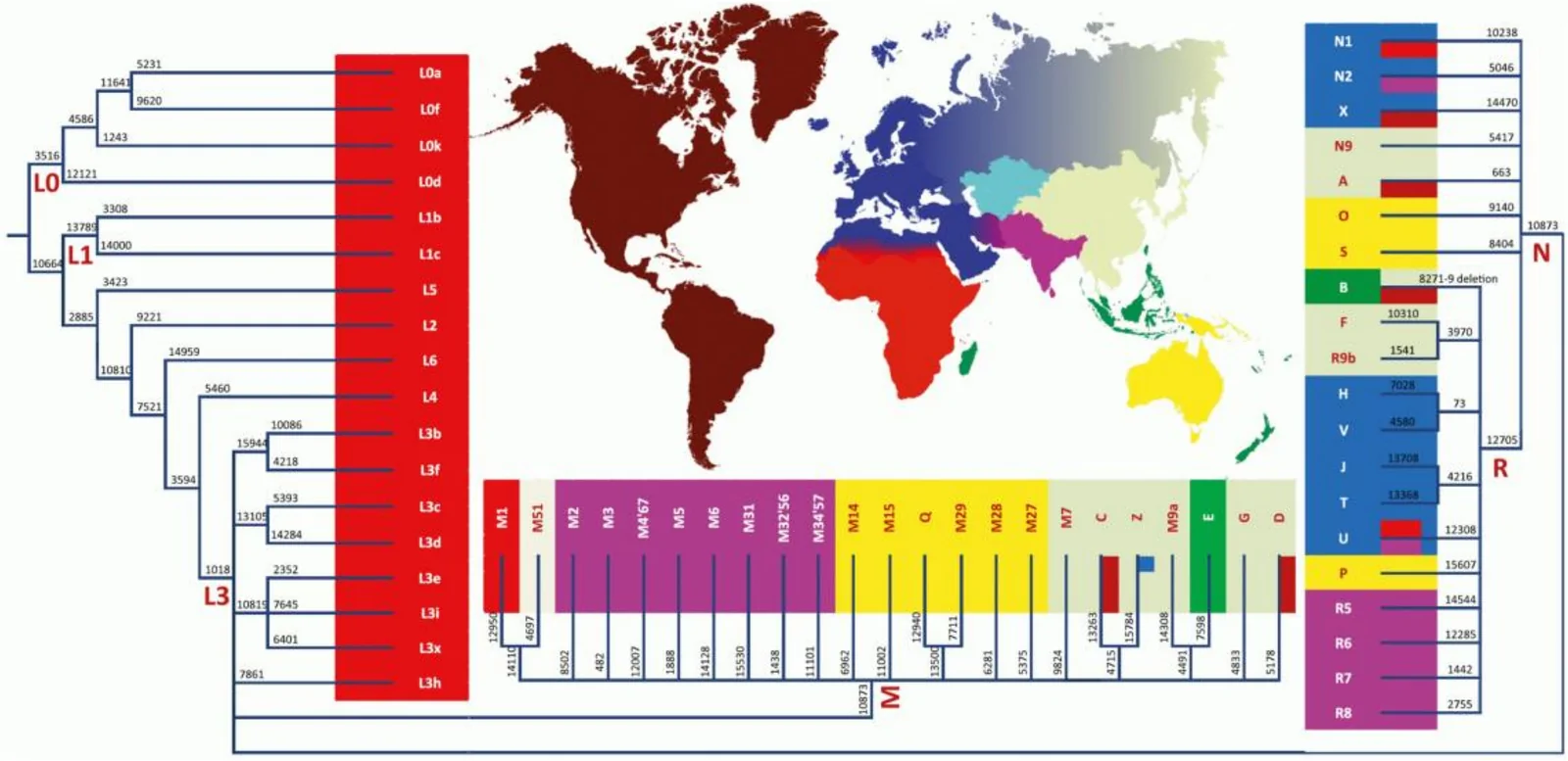
**mtDNA haplogroup tree and distribution map.** Haplogroup labels are reported according to the http://www.phylotree.org/ nomenclature (van Oven M (2009)). Only a single branch defining marker, preferably from the coding region, is shown. The main geographic features of haplogroup distribution are highlighted with color*.*

Certain algorithms in several tools, including Haplogrep3,^38^, ^39^, ^40^ HaploFind,^41^ MitoMaster,^42^ Phylotree,^37^ and MitoTool,^43^ are used to classify mtDNA haplogroups.

These tools rely on selecting a phylogenetic tree to infer haplogroups. Over time, these tools have been optimized to bypass oversights in haplogroup classification. Additionally, haplogroup classification has been enhanced throughout the years by modifying phylogenetic trees.^19^, ^37^

It's also critical to note that the mitochondrial background (haplogroup) from which a mtDNA variant originated implies its effects. The classic example, haplogroups N and M, both contain the mutant 3394C (Y30H). The mutant 3394C (Y30H) in haplogroup M is necessary for populations living at high altitudes to adapt to high altitude. However, in haplogroup N, it increased the penetrance of milder complex I variants and decreased complex I activity, which is believed to be linked to Leber hereditary optic neuropathy (LHON).^44^

In several studies, scientists have found haplogroup-specific susceptibility to certain disorders.^45^, ^46^ For example, type 2 diabetes is associated with the Asian haplogroup N9a,^47^ and autism is associated with some European mtDNA haplogroups.^48^

#### 2.2.3 mtDNA variants

There are two main types of mtDNA variants: *homoplasmic and heteroplasmic***.** Normally, an individual contains only one type of mtDNA termed homoplasmy**.** Among the thousands of copies of mtDNA that exist within a cell, some will undoubtedly experience mutations. Thus, some of the mutated copies of the genome may be passed on to subsequent generations, resulting in a mixture of distinct mitochondrial DNA (mtDNA) genomes. However, before a mature oocyte forms, a genetic bottleneck occurs that maintains homoplasmy as the norm.^49^ The bottleneck reduces the chance of passing on a mixture of wild-type and mutant mtDNA genomes by allowing only a small number of mtDNA molecules to pass through the oocyte during its formation.^50^ Heteroplasmy refers to the presence of multiple mtDNA variants within a single cell. This occurs when a mother transmits both a normal (wild-type) and a mutated version of her mitochondrial genome to her offspring. An individual will therefore possess two versions of mtDNA, usually differing by one base.^51^ However, instances of heteroplasmic mtDNA differing at two or even three positions have been reported.^52^

Homoplasmic variations are further classified into three types: *recent deleterious maternally inherited, somatic, and ancient adaptive variants.* The ancient adaptive variants are those required for adaptation to different environmental conditions during migration. So, these variants are suggested to be relatively specific to each geographic location and could thus be used as mtDNA ancestry markers.^53^

Somatic variants (base substitutions or deletions) accumulate over time in the mtDNA due to both genetic and environmental factors.^54^ It has become increasingly clear that single-nucleotide substitutions, single-base insertions, and single-base deletions are the most common types of mtDNA mutations observed, and that most of us carry a significant number of low-level mtDNA variants in our cells.^55^ Previously, the most frequently detected somatic mtDNA mutations were large deletions that result in smaller, circular mtDNA molecules.^55^, ^56^ Recent advances in next-generation sequencing techniques for whole-genome analysis have enabled the analysis of mtDNA mutational spectra across many tissues in great depth. The findings revealed that somatic mtDNA mutations increased with aging.^55^, ^57^, ^58^, ^59^, ^60^ The most common mutations found in these investigations are transition mutations, which are correlated with deamination of cytidine and adenosine, or misincorporation by DNA polymerase γ as the main mutagenic processes in mtDNA.^58^, ^59^, ^60^

Overall, findings disputed the idea that oxidative damage is a primary factor in mtDNA mutagenesis. They suggested that the majority of accumulated mtDNA point mutations are due to replication errors and/or spontaneous base hydrolysis.^55^

*A heteroplasmic variant***,** which is present in a subset of the mtDNA molecules, can arise in both somatic and germline cells. However, during the replication process (meiotic or mitotic cell division), there is a random shift in heteroplasmic allele frequency, resulting in a variation in the proportion of mutant-to-normal mtDNA in different tissues and organs.^28^, ^61^ The process is not fully understood, but nuclear genes may influence its regulation. Pathogenic heteroplasmic mitochondrial variants are those associated with genetic disorders.^62^ According to reports, over 600 distinct point mutations are currently linked to various human diseases (source: Mitomap.org).

Recently, with the rapid development of high-throughput sequencing technologies, it has become possible to sequence the Whole genome, leading to a better understanding of mitochondrial diseases. There are hundreds of pathogenic homoplasmic and heteroplasmic mitochondrial variants reported through next-generation sequencing.^63^ In 1988, a disease-linked mtDNA mutation was first discovered: mutation 11,778 G > A linked to Leber hereditary optic neuropathy disease.^64^ Although the prevalence of mtDNA variations linked to diseases has been extensively studied in modern populations, their systematic investigation in ancient populations remains limited. For collecting data on mtDNA variations, including their clinical significance and current population frequencies, several publicly accessible databases are available^63^:

• The GenBank database is an annotated collection of publicly accessible DNA sequences and was used to gather data on variant frequencies in modern populations.^65^
• MitoMap is a database of the human mitochondrial genome that includes 14,383 SNVs, 49135 full-length sequences, and 72,235 control region sequences.^66^ If a variant satisfies the criteria set forth, it is classified as “confirmed pathogenic”.
• MITOMASTER is used to analyze human mitochondrial SNVs or nucleotide sequences.^42^ The MITOMAP-MITOMASTER website is a comprehensive catalogue of mitochondrial variants, which is publicly available at https://www.mitomap.org/MITOMAP.
• Mito TIP is an in silico tool that predicts the probability that a novel single-nucleotide variant will cause disease.^67^
• HmtVar is a variant-focused database that provides information on mtDNA variability and pathogenicity.^68^

Several ancient DNA studies have reported mtDNA variants that have been associated with disease or classified as pathogenic based on evidence from modern populations. For example, 25 Thracian mtDNA samples,^69^ dating from 3000 to 2000 BCE, were whole-genome sequenced. Of them, 608 mtDNA variants were found, and only one (m.15326 A > G (rs2853508)) was identified as likely pathogenic and linked to breast cancer.^70^ The MitoMap database (2019, update nr. 3) was used to estimate the frequency of this variant across all analyzed samples, yielding a 0.98 population frequency. The high frequency means that this variant is common and thus probably not disease-related.^66^

In another study, analyzing ancient mtDNA identified six variants as “confirmed pathogenic” in contemporary patients. The very first of these was found in a Neolithic sample from 5800 to 5400 BCE that carried the MT-TS1 gene mutation m.7510 T > C. A sample from a Bronze Age site in Germany (2029–1911 BCE) revealed another variant, m.3243 A > G (rs199474657), which is one of the most prevalent mtDNA variants that putatively associated with mitochondrial disease.^63^

### 2.3 Sequence miscoding and postmortem damage in ancient remains

Miscoding lesions are a distinctive feature of aDNA studies.^71^ The observed miscoding lesions have been suggested to result from two mechanisms. The first is due to frequent errors by DNA polymerase during PCR amplification, in which incorrect nucleotides are accidentally incorporated. Such errors can lead to modifications of template molecules during the early stages of PCR, when the initial concentration of template molecules is low. The second mechanism is the creation of errors due to postmortem biochemical damage to the initial template molecules.^71^

The moment a cell dies, damage to the DNA molecule begins, with reactive oxygen species and other chemicals, as well as biomolecular processes—particularly the uncontrolled activity of lysosomal nucleases—blaming.^72^ Because compartmentalization keeps these destructive enzymes under control throughout the cell's life, endogenous DNA remains intact within the cell. Furthermore, during cell life, various repair mechanisms can correct errors in the DNA sequence and prevent endogenous DNA degradation caused by reactive oxygen species and other chemical agents.^73^

A variety of different types of damage can occur when damage to the DNA molecule is not detected during cell life or happens after death, including single- and double-stranded breaks, oxidative or hydrolytic lesions, and cross-linking of DNA to other biomolecules such as proteins, sugars, fatty acids, RNA, and DNA.^74^ According to earlier research, different types of damage can affect the DNA molecule and often result in sequence variation across duplicates from the same person.^2^

Both hydrolytic and oxidative attacks on the phosphodiester backbone, as well as enzymatic processes, are responsible for the extreme reduction in the size of ancient DNA sequence fragments.^75^ Apurinic/apyrimidinic (AP) sites are created when the N-glycosyl bond, which connects the deoxyribose sugar to the nitrogenous base, is hydrolytically attacked (Fig. 3).

**Fig. 3:**
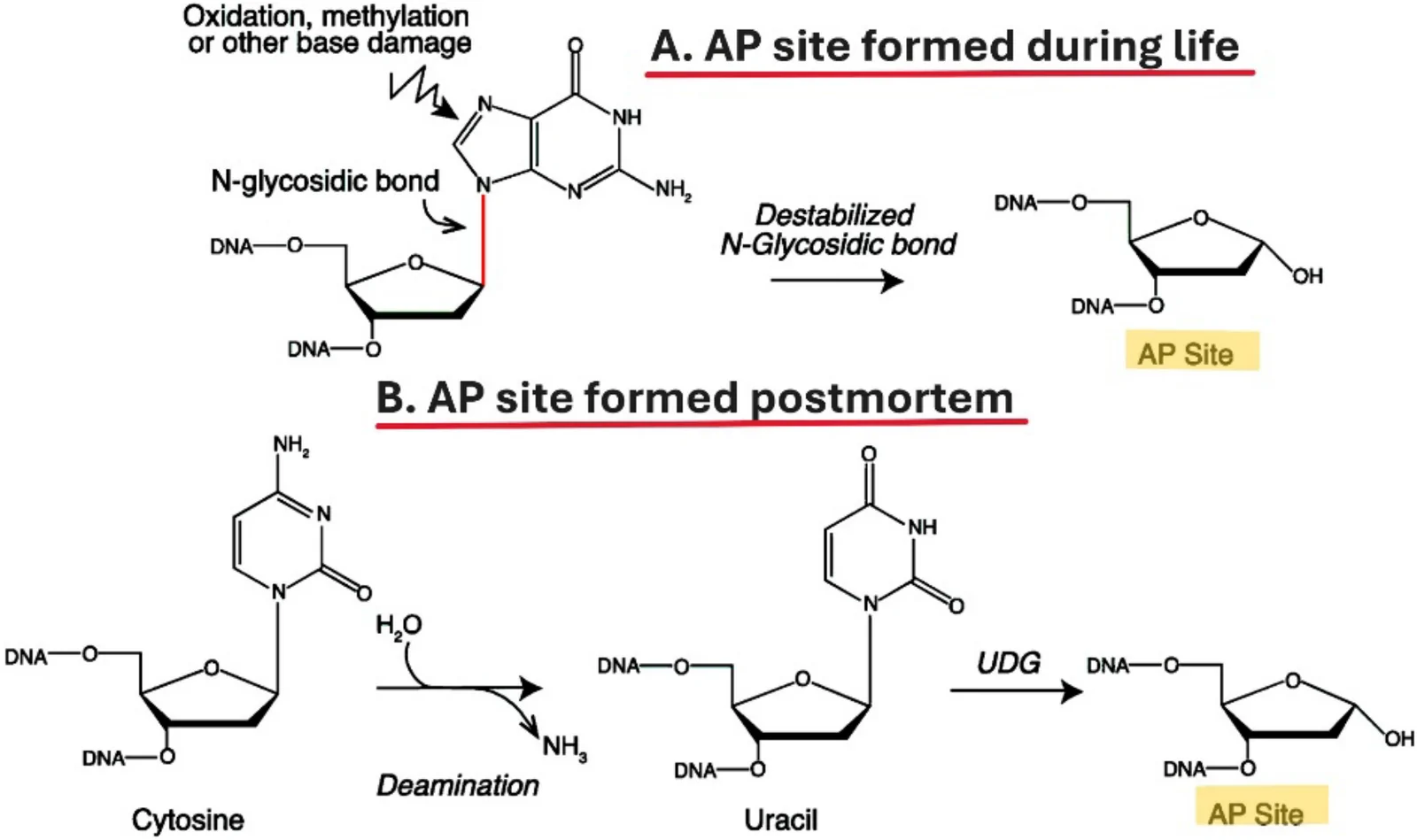
**Mechanisms of AP site formation**. A. Destabilization of N-glyosidic bond after base damage generates AP site. B. Spontaneous or catalyzed deamination of cytosine generates uracil. Subsequent action of UDG produces an AP site.

During in vitro amplification, *AP sites* themselves can cause strand breakage or inhibit DNA polymerase activity.^75^ The study of the processes that impact biological material after death is known as taphonomy.^76^ Numerous variables, such as time since deposition, the taphonomy of the environment in which the remains are recovered, and the storage conditions of the samples, could affect the degree of depurination or depyrimidination noticed within a particular sample.

DNA preservation or degradation over time is affected by i) biotic factors (bioturbation, soil bacteria, fauna, fungi, and other flora). ii) abiotic factors (radiation, physical damage, chemistry, pH, temperature, oxygen content, water content, soil type, porosity, weather processes, and humic substances). The formation of cross-links between DNA and other molecules will be broken down, altered, and catalyzed by these taphonomic variables.^77^

In addition to producing AP sites, oxidative damage can cause mutagenic effects, leading to base damage or DNA lesions in the DNA backbone. This can lead to a fragile strand that may break, thus contributing to the fragmentation process. Reactive oxygen species, particularly hydroxyl radicals and hydrogen peroxide, are the main offenders behind oxidative damage.^72^ Although oxidative attack can occur at the double bonds of both purines and pyrimidines, resulting in ring fragmentation, the most common outcome of DNA oxidation is the production of 8-hydroxyguanine from guanine, which preferentially base pairs with adenine instead of cytosine, causing the G/C bond to be transversed to a T/A bond during the subsequent amplification.^78^, ^79^ The attack can also occur within the deoxyribose molecule, breaking the sugar ring.^79^ Amplification cannot proceed beyond these locations when base or sugar ring fragmentation occurs because DNA polymerase is blocked.^72^ Apart from 8-hydroxyguanine, several other oxidative damage products are produced, which can facilitate base mispairing during amplification.^79^

Base substitutions can be categorized into two types: transitions and transversions [Table 1].^80^

**Table 1:** Types of base substitutions and the principal damaging agents causing the substitutions.

Transition	Transversion
It is the substitution of a purine for another purine base or a pyrimidine for another pyrimidine.	It is the substitution of a purine for a pyrimidine or a pyrimidine for a purine.
There is one possible transition. (*C*⟷*T* or *A*⟷*G*).	There are two possible transversions. 1. Type 1 (A → C, T → G, A⟷T) 2. Type 2 (C → A, G → T, C⟷G)
In living organisms, it is less likely to cause an amino acid sequence change. So, this may remain a silent mutation.	In the living organism, it is more likely to cause amino acid sequence changes that affect the subsequently formed protein.
This type of damage is predominantly caused by Hydrolytic deamination.	This type of damage is predominantly caused by oxidative damage.
The most common type of point mutation.	This is less common than transition.

As shown in Table 1, *C*⟷*T* or *A*⟷*G* substitutions are predominantly caused by Hydrolytic deamination, a characteristic molecular damage pattern in ancient DNA.

Because DNA strands are complementary, a lesion on one strand directly results in a substitution on that strand; however, the substitution is also apparent on the complementary DNA strand following amplification. For example, depending on which DNA strand the substitution originated from, a type 2 transition can be considered either a G → A or a C → T substitution. According to reports, these type 2 transitions are the most prevalent type of postmortem damage.^80^ Previous research has provided a detailed description of the different kinds of miscoding lesions found in ancient or degraded DNA (aDNA) templates. Regardless of the temperature and moisture content of the depositional environment from which the remains were recovered, a common theme across these studies is that the primary miscoding lesion during analysis is hydrolytic deamination leading to C → T transitions.^2^, ^80^ According to certain studies using uracil-N-glycosylase (UNG), the deamination of cytosine is the only process that results in the C → T miscoding lesion.^81^ Although deamination is the primary candidate for this lesion, oxidative damage remains a plausible alternative, potentially generating the thymine analogues 5-hydroxycytosine and 5-hydroxyuracil.

This is especially true if the sample comes from an environment where hydrolytic activity is almost absent.^79^ Nonetheless, studies of ancient hair samples have revealed that oxidative damage accounts for a larger proportion of total damage (Gilbert et al., 2006), which may be due to lower water content in these samples. This shift in oxidative damage-induced misincorporations, in turn, lowers the degree of hydrolytic damage-induced misincorporations.

In addition to oxidative damage, hydrolytic damage can result in base modification via deamination of the nitrogenous base. The pyrimidines, cytosine (C), and 5-methylcytosine are the main targets of this kind of damage. These two nucleotides can be modified to yield either uracil (U) or thymine (T). When the DNA molecule is amplified, these chemical products will subsequently cause a C → T transition.^2^, ^75^, ^81^, ^82^ Deamination can also occur in purine nucleotides, adenine [A] and guanine [G]; however, this occurs to a far lesser extent. Adenine deamination generates Hypoxanthine, which preferentially base pairs with cytosine, resulting in a transition mutation from A to G. However, Guanine deamination results in Xanthine, which subsequently base pairs with cytosine.^75^ In this case, conventional base pairing resumes in the subsequent amplification step, so there is no apparent mutation in the sequence.

For a comprehensive list of all 12 potential base substitutions, see Table 1**,** where base substitutions can be divided into two “types” for both transitions and transversions.

Analysis shows that modern DNA and potential contaminant sequences exhibit a significantly lower rate of nucleotide misincorporation compared to ancient samples. In other words, errors in nucleotide incorporation are much more prevalent in ancient DNA, while modern and contaminant sequences tend to display far greater fidelity. On the other hand, 85% of the detected nucleotide misincorporations in ancient sequences are type 2 transitions.^83^ The primary cause of nucleotide misincorporations in ancient DNA is cytosine deamination. Additionally, the apparent presence of G-to-A substitutions in sequencing data doesn't directly reflect a damage signal but is introduced during the “fill-in” step of double-stranded library protocols (e.g., Meyer & Kircher, 2010). During this step, damaged overhangs are filled before adapter ligation, transforming terminal C → T lesions on one strand into apparent G → A on the complementary strand in the sequencing output. In contrast, library preparation methods that avoid this fill-in step (e.g., single-stranded library protocols^84^) preserve the authentic ancient DNA damage signature, which is characterized by C → T substitutions due to cytosine deamination at fragment termini. Thus, the observed substitution patterns strongly rely on the library preparation strategy rather than only on intrinsic DNA damage.

The changes from G to A and from C to T are distributed differently and unevenly throughout DNA molecules. It was suggested that single-stranded ends of molecules have a higher prevalence of cytosine deamination than the middle of molecules.^2^

## 3 Implications of Genetic Drift and Selection on the Mitochondrial Genome

Genetic drift and selection-driven changes in haplotype frequencies significantly affect the mtDNA molecule. It can be explained by a variety of factors, including uniparental transmission (no recombination) and the mitochondrial bottleneck process, in which mtDNA replicates from a small number of mtDNA templates, which magnifies the dilution of minor parental alleles in early development.^19^, ^85^, ^86^ A process known as Muller's Ratchet occurs when genetic drift triggers an irreversible accumulation of deleterious mutations in an asexual population's genome.^19^, ^87^ As a result, heteroplasmic drift may reduce the effectiveness of mtDNA mutations as lineage markers.

There is a distinct pattern of negative and positive selection across mutation types and heteroplasmy levels. By measuring the rates of non-synonymous to synonymous mutations and comparing their normalized ratio (dN/dS), it was found that there is strong negative selection (dN/dS close to 0) on nonsense mutations at all ages and heteroplasmy levels, confirming the rapid elimination of protein-truncating mtDNA variants. However, missense mutations may be positively selected (dN/dS > 1) at the organelle level but negatively selected (dN/dS < 1) at the cellular level, resulting in antagonistic selection landscapes.^88^ So, the dN/dS ratio should be interpreted carefully when assessing positive and negative selections because these values may not accurately reflect fitness effects when conventional population-genetic assumptions are not applicable. This is particularly relevant to mitochondrial genomes.^89^

Genetic studies would be affected by the conflict regarding the irregular occurrence of parental leakage and the maternal inheritance of mtDNA, leading to misinterpretations of mtDNA-disease risks or associated traits.^19^

## 4 Retrieval and authentication of ancient DNA

### 4.1 Challenges in DNA preservation and retrieval

Ancient DNA is naturally fragmented. Typically, short fragments of ancient DNA range in size from 10 to 150 bp.^90^ However, the possibility of incorrect mis-mappings of endogenous reads and non-endogenous (exogenous or environmental) contamination increases when ultrashort reads (≤35 bp) are included.^91^ Larger fragments, however, might be present in “younger” and better-preserved samples.^92^

Being highly abundant per cell and maternally inherited, with little or no recombination, mtDNA is believed to be better preserved in ancient remains.^21^, ^22^, ^23^ Thus, it can be used as a molecular marker to study genetic flow & phylogeny. However, mtDNA is much more susceptible to damage due to the lack of protective proteins, such as histones, and its low-efficiency repair mechanism.^93^, ^94^, ^95^

In the early Ancient DNA studies, the initial target was to sequence the hypervariable regions of mtDNA.^96^ To determine potential maternal relationships, a limited number of markers were used to analyze SNPs within the mtDNA coding region and hypervariable regions I and II (HVR—I, HVR-II), which typically define unique profiles and haplogroups.

Custom primers were used to amplify mtDNA markers, and restriction enzymes and/or Sanger sequencing were used to detect coding-region SNPs in highly degraded samples.^97^

Authentication of the results is another issue affecting the PCR approach. Exogenous human contamination can introduce a shared contaminant across multiple samples, leading to false-positive matches and erroneous kinship attributions.

Precautions are typically taken to control potential contamination. Over the past twenty years, Sanger-type sequencing (STS), especially of the control region (CR), has been the accepted method for analyzing mitochondrial DNA (mtDNA) in forensic cases involving missing persons and human identification.^98^, ^99^ However, NGS can overcome the primary limitations of STR processing by enabling whole mitochondrial genome (mtGenome) sequencing alongside superior sensitivity and throughput.

Although the control region (CR) usually receives most of the attention in forensic mtDNA analysis, using the entire mitochondrial genome (mtGenome) can yield higher discrimination.^99^ Even though STS processing works well, it is a laborious, expensive, and time-consuming procedure, especially when analyzing the entire mtGenome.^99^

To overcome many of the drawbacks associated with PCR-based methodologies, NGS techniques have been used recently to analyze aDNA.^14^ The primer-independent approach enables recovery of very short molecules and the reconstruction of target sequences from samples that were too damaged or highly fragmented for PCR analysis. With the use of high-throughput sequencing and enrichment techniques, data can be obtained even from minimal DNA.^100^ Methods have been developed to enhance the recovery and processing of short, damaged DNA fragments from ancient remains. For instance, ancient DNA-optimized extraction protocols involve silica spin columns,^101^ magnetic beads,^102^ and in-solution silica binding^103^ to maintain short DNA molecules.^104^

In contrast, several commercial capture kits are now available for targeting mtDNA using either DNA or RNA baits that tile across the mitochondrial genome, ensuring comprehensive coverage. These include Twist Mitochondrial Panel (Twist Bioscience, USA) and myBaits® Mito hybridization capture kit (Arbor Biosciences, Ann Arbor, MI, USA). These panels enable researchers to enrich the Mt. genome, perform sequencing, and analyze genetic variation, including heteroplasmy-associated variants and disease-related variants. In addition to species identification, phylogenetics, barcoding, population genetics, and other popular applications.

Another challenge is the presence of nuclear sequences of mitochondrial origin (NumtS), which can affect the analysis of mtDNA (den Tex et al., 2010; Samaniego Castruita et al., 2015; Diroma, Maria Angela et al., 2021). These sequences that have been incorporated into the nuclear genome are non-functional and non-expressive. They present a problem for mtDNA amplification and subsequent analysis as they are not currently filtered out by many mtDNA analysis tools, such as mtDNA-server.^105^ So, researchers should be aware of this potential confounding factor. On the other hand, computational techniques for NumtS identification in aDNA samples have been developed, enabling NumtS identification in aDNA sequencing.^106^ A standard procedure based on utilizing only reads mapping uniquely to a genomic reference containing the nuclear and mitochondrial genomes together proved feasible for humans and was subsequently advised in aDNA research as a means of excluding putative NumtS.^107^, ^108^, ^109^, ^110^

The accurate reconstruction of mtDNA sequences from ancient samples remains challenging due to damage, fragmentation, and/or aDNA contamination, which may result in false-positive base calls at crucial positions, leading to incorrect haplogroup designations and biased downstream analysis. Misalignments & incorrect base calling may also result from the presence of poly-C tracts in the hypervariable regions I & II when length heteroplasmy occurs.^111^, ^112^

### 4.2 Authentication of ancient DNA

The DNA fragments retrieved from ancient samples presented characteristics of authentic aDNA, such as an increase in nucleotide misincorporation at the fragment termini and short fragment length (< 100 bp). Notably, the frequency of guanine-to-adenine substitutions at the 3′ termini and cytosine-to-thymine substitutions at the 5′ termini was increased, indicating postmortem DNA damage. These damage patterns were assessed by mapDamage or complementary tools, which confirmed typical deamination signals.^113^, ^114^

A powerful tool for verifying the accuracy of whole-genome sequencing data is the mitochondrial genome. Although mitochondrial haplogroups are not unique to individuals, we can confirm the identity of a sample by comparing a complete mtDNA sequence to previous sequencing results or the expected haplotype and detect potential contamination. When the same mitochondrial DNA haplogroup is replicated across several independent sequencing experiments, it's a strong indication that the sample has been appropriately recognized, with no mislabeling or mix-up.^115^ This step is crucial for ensuring the accuracy of the results. In contrast, the presence of exogenous DNA may be indicated by the presence of uncommon or multiple mitochondrial DNA haplogroups within a single sample. Therefore, mtDNA can also be used to detect contamination. To distinguish authentic endogenous DNA from modern contaminants, researchers search for signs of DNA damage, check sequence consistency, and analyze mitochondrial DNA haplogroups. They often use specialized computational tools, such as Schmutzi^116^ to detect contamination in both whole-genome sequencing and mitochondrial data. Furthermore, mitochondrial DNA is essential for quality control. Scientists assess the depth of sequencing, the consistency of results, the completeness of the genome, and how well it matches established haplogroups for verifying the accuracy of sequencing and bioinformatics workflows. Collectively, mitochondrial DNA is an essential tool for maintaining the accuracy and reliability of whole-genome sequencing (WGS) studies.

## 5 Analytical tools & programs

Using the NGS technique made it possible to significantly increase the number of loci and individuals successfully typed, leading to the production of kinship estimates with higher resolution and a more accurate reconstruction of historical societies.^117^ Specialized bioinformatics pipelines were created to enhance the analysis of ancient molecules.^118^ Additionally, specific tools were created for the handling and analysis of molecule degradation. As an example, the damageProfiler,^119^ mapDamage,^120^ and mapDamage2.0 (RRID:SCR_001240)^121^ packages enable the description of length patterns and misincorporation. PMD tool facilitates the decontamination of ancient genomes by calculating damage patterns and identifying degraded sequences. ContamMix, ContamLD,^122^ AuthentiCT,^123^ ANGSD,^124^ and Schmutzi^116^ tools are used for estimating the contamination within human mtDNA. Schmutzi and ANGSD tools were developed to recognize and reduce the effects of contamination and damage on ancient DNA fragments and, if feasible, reconstruct high-quality ancient mitogenomes from publicly accessible raw genomic data. Schmutzi^116^ is a widely used tool for identifying the mitochondrial consensus sequence, detecting deamination patterns (endogenous aDNA marker), and estimating exogenous mitochondrial contamination. However, the ANGSD^124^ tool could reconstruct the mitogenome of a very low-quality sample that couldn't be called by Schmutzi.^125^

Mitochondrial Haplogroup classification could be performed by algorithms such as HaploGrep. HaploGrep provides an all-in-one solution for mtDNA profile quality assessment (ranging from 0 to 1) across population genetics, clinical genetics, and forensics. HaploGrep leverages the most up-to-date version of Phylotree to analyze sequences generated by both the Schmutzi and ANGSD pipelines. Access is straightforward and free via http://haplogrep.uibk.ac.at.^38^ The fully updated Haplogrep 3 release^40^ introduces several advanced features, including enhanced quality control for VCF files, reducing errors caused by poor-quality variant calls. This framework enables the rapid detection of artificial or rare mutations, as well as recombinant artifacts and missing variants. Several bioinformatics tools have been developed and applied for mitochondrial DNA analysis, as summarized in [Table 2**]**.

**Table 2:** The bioinformatic tools used for mtDNA analysis.

Bioinformatic tool	Advantages	Limitations
*Burrows-Wheeler Alignment tool (BWA)*: an alignment package that efficiently aligns short sequencing reads against a large reference sequence.	• It is a Fast and Effective tool, allowing accurate alignment of short sequencing reads. • It supports both color space reads and base space reads. • BAM files generated by BWA are compatible with most downstream tools. • The BWA aln algorithm performs particularly well with highly fragmented ancient DNA and short Illumina reads. • It supports mismatch tolerance and gapped alignment.	• It aligns reads to a linear reference genome which can reduce variant detection and coverage near the artificial breakpoint. This can be solved by using CircularMapper with BWA. • For Ancient DNA studies certain parameters need to be optimized to improve the mapping of damaged reads. • It is less effective for detecting complex mitochondrial structural variants, large deletions, and rearrangements. • It doesn't perform downstream analysis to detect heteroplasmy, estimate contamination, and generate consensus sequence.
*Phylotree:* is a powerful JavaScript library designed for visualizing phylogenetic trees which is often used by researchers to classify mtDNA haplogroups.	• It provides an updated and extensive way to classify mtDNA haplogroups which are essential for tracing maternal ancestry & population Genetics. • It is updated regularly from newly sequenced mitogenome sequences. • It includes numerous interactive features. • Researchers can find and use it through (http://www.phylotree.org).^37^ • It serves as a tool to educate others. • It is Capable of handling multiple selection categories for comparative analysis.	• It is a java-based tool which may be a barrier for some researchers. • It depends on external databases and studies that need updates often to maintain accuracy. • Loading a fully annotated mtDNA tree may slow down the browser performance.
*MITOMAP:* is a comprehensive database of mtDNA variations and their association with disease and evolution.	• It combines many details about human mtDNA, including sequence data, pathogenic mutations, population variations, and gene-gene interactions. • Easy Access. • Frequent Updates. • It has an integration with MITOMASTER, an analytical system that uses information from MITOMAP for the analysis of new information submitted by researchers.^42^, ^66^ • It serves as a tool to educate others for a better understanding of mitochondrial genomics. • It is widely used in population genetics and forensic investigations.	• It is focused on human mtDNA. • Some populations are underrepresented, which limits global applicability. • There is a need for standardization in Data interpretation. • It is difficult to predict the pathogenicity of novel mutations.
*HaploGrep:* a bioinformatic tool designed to classify mtDNA haplogroups.^38^, ^39^, ^40^	• It is a Fast and Effective tool as it utilizes Phylotree database to provide exact haplogroup classifications. • HaploGrep 3 was enhanced to support the partial FASTA sequence analysis. • It is user-friendly • It works with multiple input file formats. • It adds checks to reduce misclassification. • Haplogrep 3 has an integrated variant annotation for immediate interpretation of variants.	• It relies on the Phylotree database. The database needs frequent updates to stay useful. • Since it focuses on mtDNA analysis, it may not perform well with other DNA types. • It is difficult to handle complex heteroplasmy. • Beginners in bioinformatics tools might take some time to add new phylogenetic trees. • Its Performance depends on complete variant information.
*damage Profiler:* A tool built in Java made to identify damage patterns in ancient DNA.^119^	• It is Fast, interactive, and easy to use java- based program for coherent calculation of damage patterns in mapped NGS reads from various mapping tools. • It can be applied to single- or multi-reference mapping files, which is useful in metagenomic studies. • It is simple to integrate into existing analysis pipelines, as in nf-core/eager. • It can work with various file formats such as BAM, SAM, and CRAM. • It generates detailed outputs like damage plots, fragment length distributions, and base frequency tables • It allows customization of visualization options. • It is Available as an open-source code on GitHub.	• It Requires Java to function. • It does not perform Bayesian estimation of damage parameters, which may be required for deeper statistical analysis. • There may be limitations in the graphical interface for advanced users. • Although it is focused on analyzing damage patterns in aDNA, it requires another tool to quantify modern contamination.
*mapDamage:* a tool for analyzing damage patterns in sequences generated from aDNA using next-generation sequencing platforms.^120^, ^121^	• The tool is useful for distinguishing ancient DNA from modern contaminants based on cytosine deamination. • It provides useful summary tables and extracts information for authenticating aDNA based on DNA damage signatures. • It uses Bayesian statistical models to determine important characteristics of aDNA molecules. • It can work well with different DNA library protocols. • It allows quantitative comparisons of damage levels across different samples. • It can be accessed freely through GitHub.	• Users need to install SAMtools and R. This setup may not work well for everyone. • Although it is focused on analyzing damage patterns in aDNA, it requires another tool to quantify modern contamination. • It offers few options for customizing output plots. • It can slow down processing of large datasets.
*PMDtools* is a software tool designed to detect postmortem damage patterns and hence decontaminate the ancient genomes.	• It can distinguish ancient DNA from modern contamination. • It uses a likelihood framework incorporating damage patterns, base quality scores, and biological polymorphisms for the accurate identification of aDNA. • It allows users to customize their filtering options. • It can be integrated into existing pipelines and supports SAM-formatted input files. • free-to-use software	• It relies on external programs like SAMtools to work with BAM files and uses R to create plots that might not be suitable for non-technical users. • It requires an input BAM file with MD tags, a specific feature in the output file of many aligners. • It specializes in detecting ancient DNA damage. • It doesn't quantify contamination levels.
*ContamMix:* is a tool used for estimating contamination in ancient mtDNA using a mixture-model approach.	• It uses Markov Chain Monte Carlo (MCMC) methods to estimate contamination levels with high accuracy. • free-to-use software. • It generates clear documentation, including plots for contamination estimates, that helps in the interpretation. • It can be integrated into existing pipelines and works with various sequencing data formats like BAM & FASTA files.	• It might not be accurate with Low-Quality Consensus Sequences. • It can take time to learn as it is command-line based. • The method assumes that contamination is less than 50%, which might not always be the case. • It may ignore sites with ambiguous characters. • The tool focuses on estimating mtDNA contamination and not the nuclear DNA contamination.
*ContamLD:* is a tool designed to estimate nuclear aDNA contamination by measuring the breakdown of linkage disequilibrium (LD) due to the introduction of contaminant DNA.^122^	• It provides accurate estimates of contamination rates with low standard errors, even with low levels of contamination (<10%). • It can effectively distinguish between authentic ancient DNA and contaminant DNA by measuring the breakdown of LD • Its contamination estimates are based on aDNA damage patterns. • It is an open-source tool. • It is applicable for both sexes, unlike ANGSD which is applicable for X-Chromosome only.	• It requires Python 3 and R, as well as other tools like PMDtools and SAMtools, which might not be ideal for all users • It can be time-consuming as it requires many pre-processing steps. • Its accuracy decreases with low coverage (<0.5×). • It specializes in analyzing ancient DNA. • It may require significant computational resources, especially for low-coverage whole-genome shotgun sequences. • It is hard to learn.
*AuthentiCT:* a command-line tool designed to estimate the proportion of present-day DNA contamination in ancient DNA datasets generated from single-stranded DNA libraries.^123^	• It relies on patterns of post-mortem damage to make an accurate estimate of contamination in single-stranded DNA (ssDNA) libraries. • It doesn't require high data and can measure contamination with just 10,000 mapped sequences which is useful for poorly preserved specimens. • It is a free-to-use tool • It is an easy-to-use tool.	• It needs Python 3.6 and several dependencies. • It specializes in libraries of ssDNA and it is not suitable with UDG-treated samples or double-stranded DNA (dsDNA). • It requires SAM file with an MD field as an input file.
*ANGSD: is software* developed for analyzing NGS data.^124^	• It can handle large datasets efficiently. • It can handle different input formats, such as BAM and CRAM. • It is ideal for low-depth sequencing data as it works with genotype likelihoods instead of called genotypes. • It can perform population genetic analyses, association mapping, and summary statistics • It allows you to process multiple tasks at once and get results quickly and efficiently. • It is a Free and open-source software.	• Results can be changed based on quality filtering. • It is not recommended for high-depth data as it may be less accurate. • It doesn't support indels in any analysis. • It isn't easy to learn as it is a Command-line interface. • It has mostly been tested and proven to work on GNU/Linux systems, but it can also compile on OSX with some limitations.
*Schmutzi:* an iterative approach to estimate present-day human contamination in aDNA datasets, with a focus on reconstructing endogenous mtDNA.^116^	• It uses patterns of post-mortem damage for accurate contamination estimation. • It can rebuild the endogenous mitochondrial genome, accounting for contamination and deamination. • It uses a Bayesian maximum a posteriori (MAP) estimate, which gives strong and reliable results. • Free and Open Source. • It generates detailed Documentation.	• It relies on External Tools • It may underestimate contamination levels if there are multiple present-day human contaminants. • Accuracy might not be achieved in low-coverage samples. • It is designed for mtDNA analysis and might not work well for other kinds of DNA analysis. • It may require significant computational resources, especially for large datasets. • It runs better with Linux and macOS.

Next-generation sequencing technology evolves so quickly that bioinformatics pipelines need to be updated frequently. The main steps of the workflows are well known. However, new tools and software are continuously being developed. Tools and software updating will have significant impacts on data analysis, altering what researchers have found. Choosing the appropriate tool depends on how much detail you need while considering available time, funds, and computing resources.

## 6 Ethical considerations in ancient human remains research

Ethical considerations in ancient human remains are the principles used to protect individuals, communities, researchers, and scientific knowledge. Such considerations should be applied across all stages, from initial recovery to data analysis to retain their cultural and archaeological context. While ancient human remains serve as a scientific resource for biological and archaeological information, they represent the material remains of individuals and may possess spiritual, or cultural importance for descendant communities.

Consequently, an ethical framework for the scientific study of ancient human remains has been proposed, assessing the relationship between ancient human remains and researchers.^126^ It keeps a balance between posthumous rights and the advancement of scientific understanding, the level of invasiveness and appropriateness of sampling and processing, as well as the relevant transport and storage, and the appropriate management of data generation, interpretation, and publication. Such considerations are highly relevant to ancient DNA studies, in which analysis may require destructive sampling of skeletal remains or teeth, highlighting the importance of preserving the remaining specimens while limiting sample removal, and ensuring proper handling and storage throughout the research process.

Consistent with these considerations, Current ethical frameworks have recognized the importance of clarity, proper handling of human remains, and active collaboration with archaeologists, curators, and communities with cultural or ancestral connections to the remains. Such considerations are highlighting the importance of recognizing ancient human remains as representing individuals rather than treating them solely as biological research resources.^127^, ^128^, ^129^, ^130^, ^131^

Before undertaking sampling, the physical structure and condition of the remains should be documented, and all collected samples should be appropriately labeled, securely stored, and systematically tracked. Destructive sampling in biomolecular investigations should be scientifically supported and should involve the minimum amount of material necessary to achieve the study objectives. Preserving DNA extracts and sequencing libraries, when allowed, may help minimize the need for repeated destructive sampling in future studies.

## 7 Conclusion

This review addresses the role of mitochondrial DNA in the analysis of ancient samples. The major strength of utilizing mtDNA over nuclear DNA (nuDNA) is its higher retrievability potential with no recombination, due to its high copy number in the cell. Despite this, there are certain restrictions and drawbacks to limiting the study of human remains to mtDNA analysis only.

Advances in laboratory protocols and sequencing technologies have made it possible to more effectively retrieve mtDNA sequences from skeletal remains found in archaeological sites with improved data quality and authenticity, even from highly degraded samples.

In paleogenomic studies, the standard nowadays for mitochondrial DNA analysis is to recover the whole mitochondrial genomes either through deep sequencing for well-preserved samples or through targeted capture enrichment for more degraded samples.

Although contamination and postmortem damage remain as existing challenges, advances in methodology and integrative approaches are expected to improve the resolution and reliability of mtDNA-based inferences.

## CRediT authorship contribution statement

**Dina W. Ibrahim:** Conceptualization, Writing – original draft, Investigation, Visualization, Writing – review & editing. **Haidan Mostafa El-Shorbagy:** Writing – review & editing. **Ismail A. Abdelhamid:** Writing – review & editing. **Yehia Z. Gad:** Supervision, Conceptualization, Writing – review & editing.

## Declaration of competing interest

The authors declare that they have no known competing financial interests or personal relationships that could have appeared to influence the work reported in this paper.
